# Supplementary material for: The interactome of intact mitochondria by cross-linking mass spectrometry provides evidence for coexisting respiratory supercomplexes
Source: Mol Cell Proteomics. 2017 Dec 8;17(2):216–32. doi: 10.1074/mcp.RA117.000470 (PMC5795388; doi:10.1074/mcp.RA117.000470)
Supplement: Supplemental Data [file supp_RA117.000470_133922_0_supp_23978_fzffwf.zip › spectra_annotation/mito_spectra_annotation/52-1-6-1-3-1.pdf]

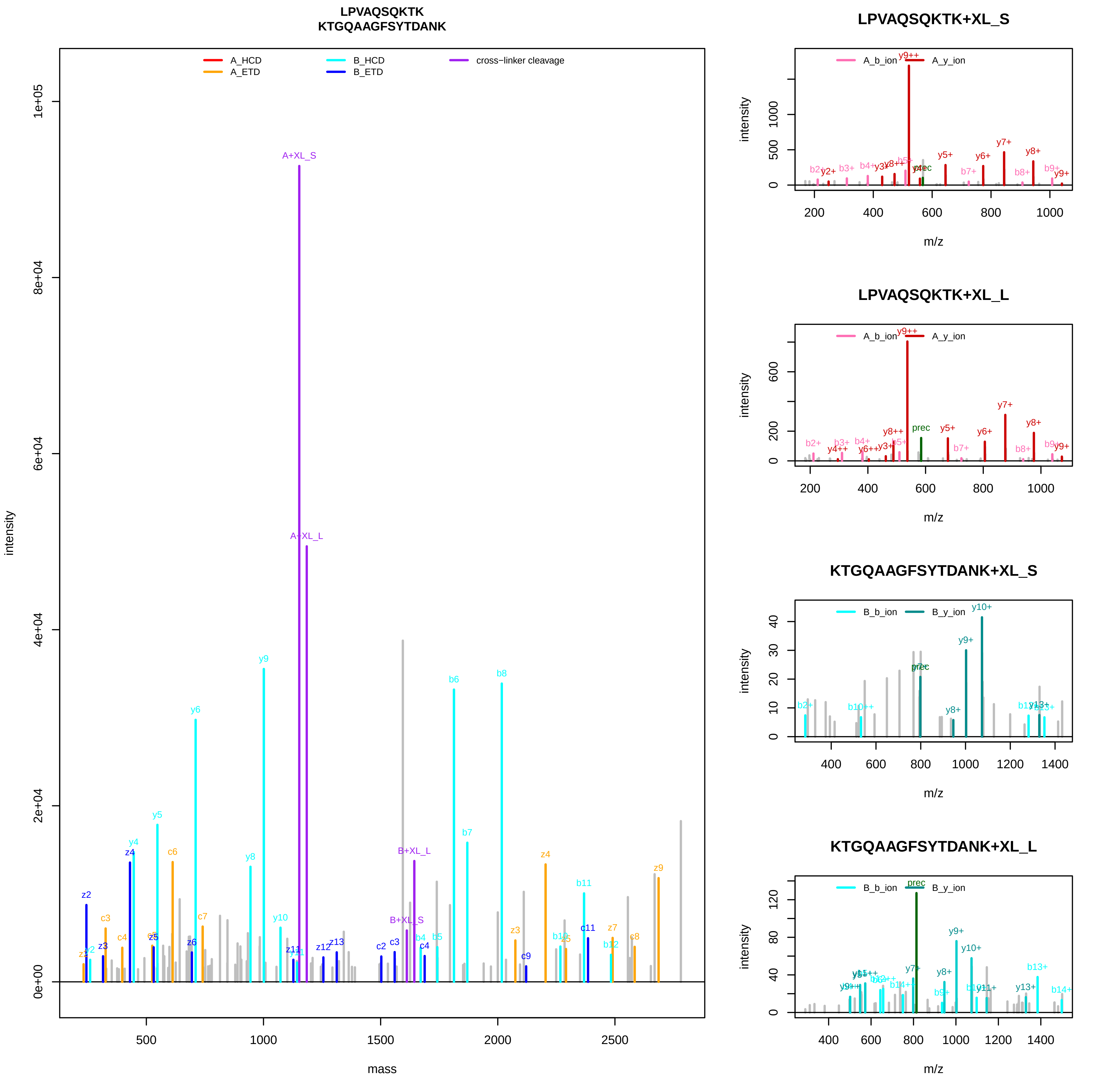

LPVAQSQKTK
KTGQAAGFSYTDANK
A_HCD
A_ETD
B_HCD
B_ETD
cross−linker cleavage
A+XL_S
A+XL_L
B+XL_L
B+XL_S
intensity
mass
0e+00
2e+04
4e+04
6e+04
8e+04
1e+05
500
1000
1500
2000
2500
y9
y6
y5
y4
z4
y8
c6
c7
z2
c3
c4
z3
z6
y10
z11
z12
z13
c2
c3
c4
b4
b5
b6
b7
b8
z3
c9
z4
b10
z5
b11
c11
b12
z7
c8
z9
LPVAQSQKTK+XL_S
A_b_ion
A_y_ion
y9++
intensity
m/z
LPVAQSQKTK+XL_L
KTGQAAGFSYTDANK+XL_S
B_b_ion
B_y_ion
y10+
y9+
prec
KTGQAAGFSYTDANK+XL_L
